# Exhibition by the Drug, Chemical, and Allied Trades

**Published:** 1895-09-21

**Authors:** 


### EXHIBITION

### BY THE DRUG, CHEMICAL, AND ALLIED TRADES.

This exhibition, organised by the *British and Colonial Druggist*, took place at the Agricultural Hall, Islington, last week, and there were many interesting exhibits. The Pompadour Band (Miss Eleanor Clausen's orchestra of young ladies) played during the four days the exhibition was open. A conference of chemists was held on Tuesday upon "Anti-Cutting"; a lecture was given by Dr. S. Rideal upon "Filtration, with Special Reference to Modern Filters," on Wednesday evening; there was a drill and ambulance display by the St. Clement's Ambulance Brigade on Thursday; and, by way of conclusion, the Exhibitors' Association gave a smoking concert on Friday, the last day of the exhibition.

The stands were well arranged, and we must sound a note of special praise over the clear numbering of each stall, a deficiency in this matter being so frequent a cause of complaint in many exhibitions of the kind, where the unhappy visitor wanders up and down, sadly seeking to reconcile the information in his catalogue with the chaos of apparently un-numbered stalls on every side.

James Lyle and Co. were showing a neat little bed-table of polished walnut wood among a variety of other exhibits. The Catley Abbey Natural Seltzer Water Company were to the fore with their mineral water, for which much use is claimed in cases of gout and rheumatism, and as a "British product" possesses a special interest. Soaps of all kinds were to be seen on the stall of Edward Cook and Sons; and next door to these Robinson and Sons had an exhibit of antiseptic and absorbent surgical dressings, cotton wools, and gauzes. This firm were also exhibiting the now well-known pill-box and bandage shoots of Reynolds and Branson, together with the "Non-run-away Bandage," a clever little contrivance for keeping bandages from unwinding themselves while being adjusted The Condal Water Company showed their natural mineral water; Parke, Davis, and Co. had specimens of a variety of food preparations, and medicinal specialities. Ointments, powders, and disinfectants jostled each other on the stall of Jeyes' Sanitary Compounds Company. Leslies (Limited) had all kinds of plasters, strappings, mustard leaves, &c.; sponges of all kinds, shapes, and sizes were shown by Cresswell Brothers and Schmitz; the Volcanic Aeration Company demonstrated their apparatus specially adapted for hospital and institution requirements. A new preparation, "Jerezcona Wine," a combination of "a fine old sherry with the active principle of the cinchona bark," was exhibited by Hertz and Collingwood. Burroughs, Wellcome, and Co. had an imposing stand, furnished with specimens of their many well-known preparations, and the medicine chests and cases with which so many Arctic and other expeditions have been supplied. Soap and perfumery were represented at the stall of Sharp Bros.; elastic and other bandages, belts, and trusses at that of Vincent Wood; Æsculap, Carlsbad, Vichy, and other waters were exhibited by Ingram and Royle (Limited); the Sanitas Company had innumerable disinfectant and deodorising preparations; "Mitchell's Castor Oil" was shown by the British Castor Oil Company (Limited), and many drugs and preparations by Thomas Christy and Co.; Armour and Co., of Chicago, had many varieties of their well-known meat extracts and peptones; Oppenheimer and Co. exhibited their "palatinoids," and soluble hypodermics in the form of tiny pellets immediately soluble in cold water, and many other preparations; "Papain," B. Kühn's new "Vegetable Digestive Ferment," was on exhibition; Forbes, Abbott, and Lennard showed disinfectants, &c.; feeding bottles and cups and surgical instruments, together with many nurses' necessaries, were brought to notice by Hockin, Wilson, and Co.; and lastly, we must not omit to mention the "Terrol" Company, who showed their speciality of that name. Of many other exhibits space forbids further mention.

